# Delirium and health-related quality of life in severe COVID-19 survivors

**DOI:** 10.1192/j.eurpsy.2022.794

**Published:** 2022-09-01

**Authors:** L. Chorão, S. Martins, A.R. Ferreira, J. Fernandes, T. Vieira, L. Fontes, N. Reis, A. Braga, I. Coimbra, J.A. Paiva, L. Fernandes

**Affiliations:** 1 Faculty of Medicine of the University of Porto, Fmup, Porto, Portugal; 2 Faculty of Medicine - University Porto, Department Of Clinical Neuroscience And Mental Health And Center for health technology and services research (cintesis), Porto, Portugal; 3 Centro Hospitalar Universitário São João (CHUSJ), Intensive Care Medicine Department, Porto, Portugal; 4 Faculty of Medicine - University Porto, Department Of Medicine, Porto, Portugal; 5 Psychiatry Service, Centro Hospitalar Universitário De São João, Porto, Portugal

**Keywords:** Covid-19, Quality-of-life, Critical illness, delirium

## Abstract

**Introduction:**

Severe COVID-19 survivors experience long-term neuropsychiatric morbidity, particularly those who developed delirium, with a negative impact on health-related quality of life (HRQoL).

**Objectives:**

To identify the cases of delirium in severe COVID-19 patients and to describe its association with post-hospital discharge HRQoL.

**Methods:**

In the context of the longitudinal MAPA project, we included adult patients (≥ 18 years old) admitted with COVID-19 to the Intensive Care Medicine Department (ICMD) of a Portuguese University Hospital (October 2020-April 2021). Exclusion criteria were: ICMD length of stay ≤24h, terminal illness, major auditory loss, or inability to communicate at the time of assessment. Delirium during ICMD stay was ascertained based on patients’ clinical records. HRQoL was evaluated using the 5-Level EQ-5D questionnaire (EQ-5D-5L), at a scheduled telephone follow-up appointment on average 1-2 months after hospital discharge.

**Results:**

Overall, 124 patients were included with a median age of 62 (range: 24-86) years, being mostly male (65%). About 19% had delirium, 42% were deeply sedated and 43% required invasive mechanical ventilation. Most survivors reported problems on the EQ-5D-5L domains: usual activities (85%), mobility (73%) and anxiety/depression (65%). Patients with delirium reported more pain/discomfort (75%vs46%; p=0.011) and considerably anxiety/depression (83%vs60%; p=0.032).

**Conclusions:**

These findings pointed that COVID-19 patients who experienced delirium reported worse HRQoL, regarding pain/discomfort and anxiety/depression. This study highlights the importance of not only prevention but also early screening of delirium during hospital stay, as well as the crucial role of the timely interventions at discharge, in order to minimize delirium long-term impacts.

**Disclosure:**

No significant relationships.

